# Pneumocephalus as a Complication of Transsphenoidal Surgery for Pituitary Adenoma: A Case Report

**DOI:** 10.7759/cureus.3104

**Published:** 2018-08-05

**Authors:** Subhanudh Thavaraputta, Eman N Attaya, Joaquin Lado-Abeal, Ana M Rivas

**Affiliations:** 1 Department of Internal Medicine, Texas Tech University Health Sciences Center, Lubbock, USA; 2 Department of Radiology, University Medical Center, Lubbock, USA; 3 Division of Endocrinology/Department of Internal Medicine, Texas Tech University Health Sciences Center, Lubbock, USA

**Keywords:** pituitary adenomas, neuro-radiology, neurosurgery

## Abstract

Pneumocephalus (PNC) is a rare complication of transsphenoidal surgery that can result from cerebrospinal fluid (CSF) leak, allowing air entry into the CSF. We report the case of a 49-year-old female patient who presented to the emergency department three weeks after a transsphenoidal pituitary tumor resection, with symptoms of generalized throbbing headache associated with nausea. The patient was alert and oriented without any focal neurological deficit. A head computed tomography (CT) scan showed air in the subarachnoid space and ventricles. She was admitted to the hospital and was initially treated conservatively. However, her symptoms persisted, and a repeat head CT scan demonstrated worsening PNC. She then underwent lumbar drain placement and sellar floor repair. Her symptoms resolved postoperatively.

When PNC results in intracranial hypertension, it is referred to as tension PNC, a complication that can be fatal. Conservative treatment involves analgesics and therapy for intracranial hypertension. Surgical intervention to decrease intracranial hypertension and repair the CSF leakage may also be necessary.

## Introduction

Transsphenoidal surgery for pituitary adenomas is the standard surgical approach for most pituitary adenomas. Despite the improvement in the technique and availability of more modern tools, complications can still occur in around one percent of all cases [1]. Pneumocephalus (PNC), the presence of air within the cranial cavity, is rare but potentially fatal resulting from the presence of cerebrospinal fluid (CSF) leak at the base of the skull [2]. PNC can progress to tension PNC in which subdural air accumulation results in mass effect over the underlying brain parenchyma. We report a case of PNC as a complication of transsphenoidal resection of a growth hormone-secreting pituitary adenoma that required treatment with CSF leak repair and lumbar spine drainage.

## Case presentation

**Figure 1:**
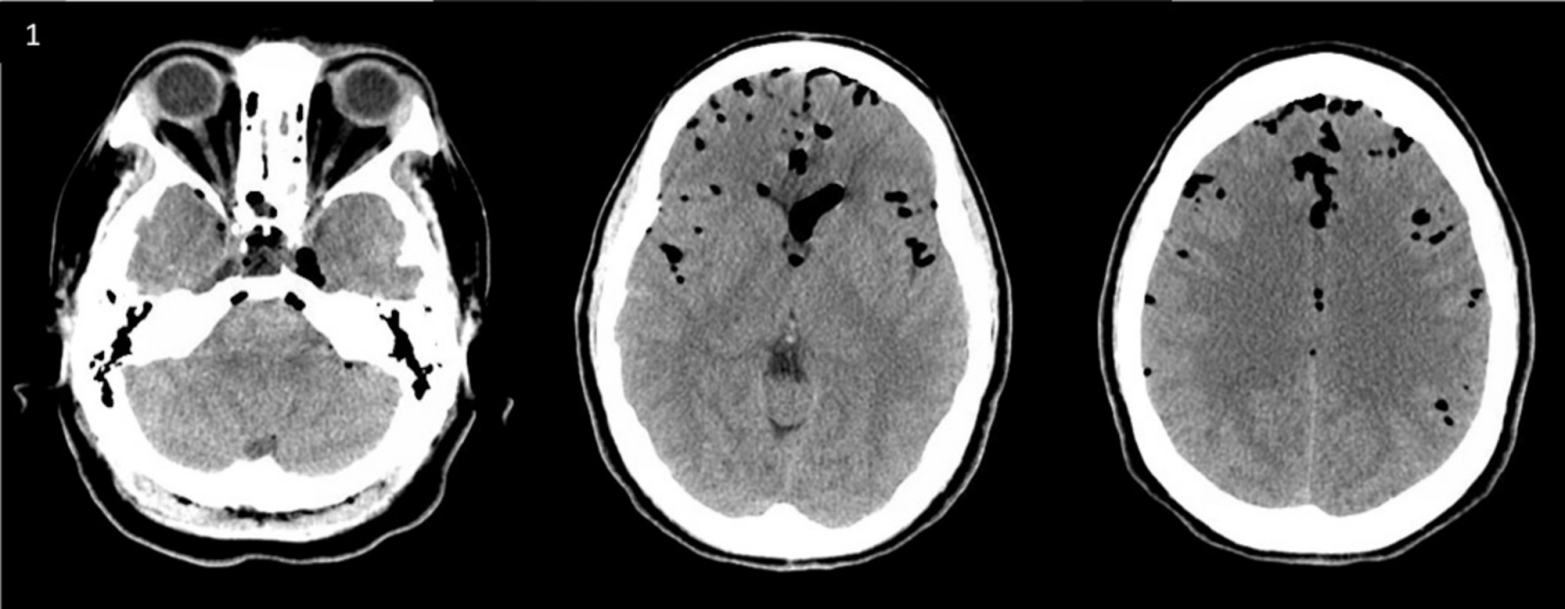
The first picture demonstrated an evidence of pneumocephalus (PNC). Axial computed tomography images of the brain on the first day in the emergency room showed diffuse PNC most pronounced in the frontal region.

A 49-year-old female with a history of acromegaly, status post-transsphenoidal pituitary resection three weeks prior, presented to the emergency room with a headache and clear nasal discharge present since the removal of a nasal splint. The patient described the cephalgia as severe, intermittent and throbbing, exacerbated by standing up and coughing and relieved by lying down and acetaminophen. On physical examination, her vital signs were normal and the results of neurological examination were normal, but a minimal clear nasal discharge was noted. A computed tomography (CT) scan of the head showed multiple air loculi in the basal cisterns, lateral, third and fourth ventricles and numerous air-filled spaces also scattered in the brain. No mass effect or midline shift was seen (Figure 1).

**Figure 2:**
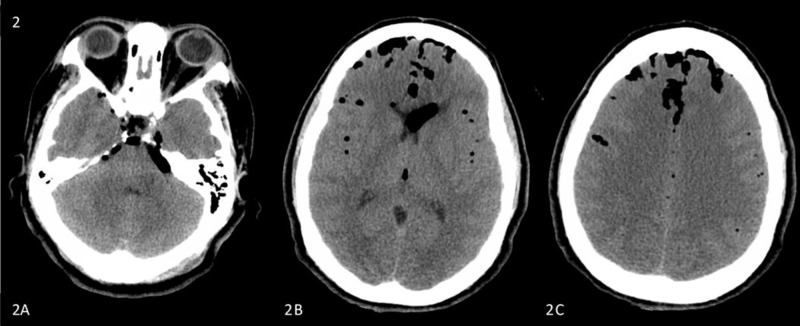
Picture demonstrated the progression of the pneumocephalus (PNC). Repeat axial computed tomography of the brain obtained a few days later: (A) Increased air in the posterior fossa causing mild mass effect on the pons. (B) and (C) Slightly increased PNC in the frontal and parafalcine regions.

Initial management consisted of bed rest in the Fowler position at 30° and instructions to avoid Valsalva maneuver such as analgesia, coughing, and sneezing. Besides the supportive treatment, the headache worsened, and a repeat CT scan showed mild increased diffuse PNC with intracranial air loculi in the parafalcine region, anterior horn of the left ventricle, posterior fossa, and left middle fossa. There was also an increased amount of air in the posterior fossa causing a mass effect on the pons (Figure 2).

**Figure 3:**
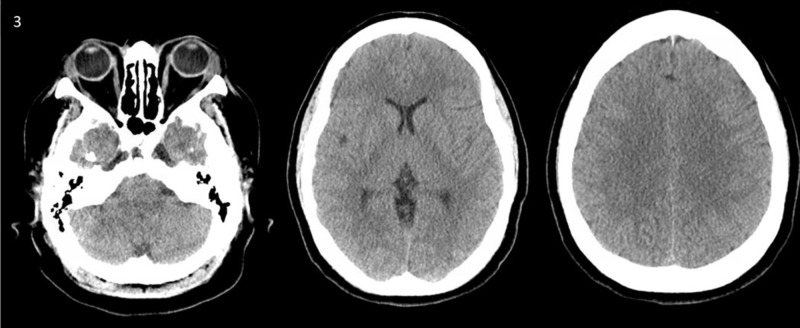
Picture demonstrated the resolution of the pneumocephalus (PNC). Follow up computed tomography scan after treatment showed resolved PNC.

She underwent a transsphenoidal endoscopic exploration of the sphenoid and sellar floor, with septoplasty and packing of the sphenoid sinus with abdominal fat graft, and with the insertion of a lumbar drain. After these procedures, she showed a significant improvement of her symptoms, the lumbar drain was removed after five days, and her headache and nasal leakage resolved. She was discharged on day 10 of hospitalization. At the time of follow up, the patient was free of symptoms, and repeat CT-scan revealed resolved PNC (Figure 3).

## Discussion

Pneumocephalus, the accumulation of air in the epidural, subarachnoid, intraventricular, intracerebral and subdural spaces, has been reported as a complication of neurosurgical and otolaryngological procedures [3]. Two mechanisms have been described to illustrate the pathogenesis of PNC. One is the  “ball-valve mechanism,” which refers to a leakage of CSF that allows air to enter from extracranial space but does not allow the air to come out. Another is the “inverted soda bottle effect,” which refers to a leakage of CSF allowing air to enter into the intracranial space to balance the pressure between the intracranial and extracranial spaces. Occasionally, PNC can result in intracranial hypertension; a phenomenon called tension PNC [4]. As PNC is an uncommon complication of transsphenoidal pituitary resection, it is sometimes not promptly recognized and can have fatal consequences. Patients who develop CSF leak as a complication of surgery should then be educated on the possibility of this complication and instructed to seek medical assistance in the setting of persistent headaches.

Currently, no consensus treatment guidelines exist for PNC. Supportive treatment involves bed rest, avoiding Valsalva maneuver such as coughing and sneezing, Fowler position of 30°, analgesia, osmotic diuresis, and antipyretics to prevent hyperthermia. High-flow oxygen through a nasal cannula and hyperbaric oxygenation therapy have been shown to be beneficial in particular cases [5-7]. This last recommendation is based on the “ball-valve mechanism,” and it is thought that creating a concentration gradient of oxygen between blood and intracranial air bubble can result in the acceleration of air resorption.

Serial imaging with CT scan is an efficient and reliable way to follow the patient’s progression [5-9]. There are no clear-cut criteria for surgical intervention but the surgical closure of a defect is usually the treatment option in patients who fail to improve with supportive treatment, presenting with progressive PNC, or that develop tension PNC [4, 8, 10].

## Conclusions

Pneumocephalus is an uncommon but potentially lethal complication of transsphenoidal surgery which can present as dull headache. As such, it could be overlooked at the time of follow up if the provider is not aware of this possibility. It is crucial to consider PNC as a differential diagnosis in patients presenting with headaches or neurological symptoms after transsphenoidal surgery.

## References

[1] DelGaudio JM, Ingley AP (2010). Treatment of pneumocephalus after endoscopic sinus and microscopic skull base surgery. Am J Otolaryngol.

[2] Ciric l, Ragin A, Baumgartner C, Pierce D (1997). Complications of transsphenoidal surgery: results of a national survey, review of the literature, and personal experience. Neurosurgery.

[3] Karavelioglu E, Eser O, Haktanir A (2014). Pneumocephalus and pneumorrhachis after spinal surgery: case report and review of the literature. Neurol Med Chir (Tokyo).

[4] Dabdoub CB, Salas G, Silveira NE, Dabdoub FC (2015). Review of the management of pneumocephalus. Surg Neurol Int.

[5] Dexter F, Reasoner DK (1996). Theoretical assessment of normobaric oxygen therapy to treat pneumocephalus. Anesthesiology.

[6] Teng J, MacIsaac RJ, Wang YY (2014). Symptomatic pneumocephalus after trans-sphenoidal surgery. J Clin Endocrinol Metab.

[7] Gore PA, Maan H, Chang S, Pitt AM, Spetzler RF, Nakaji P (2008). Normobaric oxygen therapy strategies in the treatment of postcraniotomy pneumocephalus. J Neurosurg.

[8] Siegel JL, Hampton K, Rabinstein AA, McLaughlin D, Diaz-Gomez JL (2017). Oxygen therapy with high-flow nasal cannula as an effective treatment for perioperative pneumocephalus: case illustrations and pathophysiological review. Neurocrit Care.

[9] Paiva WS, de Andrade AF, Figueiredo EG, Amorim RL, Prudente M, Teixeira MJ (2014). Effects of hyperbaric oxygenation therapy on symptomatic pneumocephalus. Ther Clin Risk Manag.

[10] Haran RP, Chandy MJ (1997). Symptomatic pneumocephalus after transsphenoidal surgery. Surg Neurol.

